# Green lacewings (Neuroptera: Chrysopidae) are commonly associated with a diversity of rickettsial endosymbionts

**DOI:** 10.1186/s40851-017-0072-9

**Published:** 2017-08-14

**Authors:** Michael Gerth, Ronny Wolf, Christoph Bleidorn, Julia Richter, Rebekka Sontowski, Jasmin Unrein, Martin Schlegel, Axel Gruppe

**Affiliations:** 1 Institute of Integrative Biology, University of Liverpool, Biosciences Building, Crown Street, L69 7ZB, Liverpool, UK; 2 Institute for Biology, Molecular Evolution & Systematics of Animals, University of Leipzig, Talstrasse 33, 04103 Leipzig, Germany; 3 Museo Nacional de Ciencias Naturales, Spanish National Research Council (CSIC), Madrid, Spain; 4 German Centre for Integrative Biodiversity Research (iDiv) Halle-Jena-Leipzig, Deutscher Platz 5e, 04103 Leipzig, Germany; 5 Chair of Zoology - Entomology, Technical University of Munich, Hans-Carl-von-Carlowitz-Platz 2, 85354 Freising, Germany

**Keywords:** *Rickettsia*, Endosymbiosis, Biological control, *Chrysoperla*, *Chrysopa*, Neuropterida

## Abstract

**Background:**

Bacterial symbionts transmitted from mothers to offspring are found in the majority of arthropods. Numerous studies have illustrated their wide impact on host biology, such as reproduction, behavior, and physiology One of the most common inherited symbionts is *Rickettsia* spp. (Alphaproteobacteria, Rickettsiales), which are found in about one-quarter of terrestrial arthropods, as well as in other invertebrates. In insect populations, *Rickettsia* spp. have been reported to cause reproductive modifications and fecundity-enhancing effects. Here, we investigated the incidence and genetic diversity of *Rickettsia* symbionts in green lacewings (Neuroptera, Chrysopidae), which are best known for their use as biological control agents against crop pests.

**Results:**

We screened 18 species of green lacewings and allies for *Rickettsia* and found the symbiont in 10 species, infecting 20–100% of sampled individuals. Strain characterization based on multiple bacterial loci revealed an unprecedented diversity of *Rickettsia* associated with lacewings, suggesting multiple independent acquisitions. Further, the detected *Rickettsia* lineages are restricted to a specific lineage (i.e., species or genus) of investigated lacewings, and these associations are stable across multiple sampled locations and points in time.

**Conclusions:**

We conclude that *Rickettsia*-lacewing symbioses are common and evolutionarily stable. The role of these symbionts remains to be identified, but is potentially important to optimizing their use in biological pest control.

**Electronic supplementary material:**

The online version of this article (doi:10.1186/s40851-017-0072-9) contains supplementary material, which is available to authorized users.

## Background

*Rickettsia* are maternally inherited Alphaproteobacteria that are estimated to be present in one of four terrestrial arthropods [1]. Historically, *Rickettsia* were best known and studied primarily because of their importance as arthropod-vectored human pathogens, causing diseases such as typhus and various types of spotted fever [2]. Bacteria with genetic similarity to these pathogens have since been discovered in many arthropods and other invertebrates [1, 3], and it has become clear that human pathogens are derived lineages deeply nested within the radiation of most invertebrate-associated *Rickettsia* [4]. In arthropods, *Rickettsia* may cause sex-ratio distorting phenotypes, i.e., male-killing [5, 6] or parthenogenesis [7, 8], but has also been associated with positive fitness effects, such as increased fecundity [9, 10] and protection from pathogens [11, 12]. For most hosts, however, the effects of harboring *Rickettsia* have not been determined.

In a recent large-scale PCR-based survey of *Rickettsia*, Weinert et al. identified many previously unrecognized arthropod-*Rickettsia* associations [3]. Among them, one individual ‘green lacewing’ (unidentified Chrysopidae from Mexico) tested positive. Furthermore, in a microscopic screen of tissues from 111 arthropod species more than 90 years ago, Cowdry discovered *Rickettsia*-like bacteria in one North American green lacewing species (*Chrysopa oculata*) [13]. Whilst these randomly sampled screens over a large diversity of hosts provide information relating to the global incidence of endosymbionts, they do not inform about the relative importance of a symbiont within a particular taxon. Targeted symbiont screens covering many species within a single taxon have thus been used to identify taxon-specific distribution patterns, and are commonly used as the first step toward deciphering the impact of a symbiont on a taxon’s biology [14, 15]. Based on these previous, isolated findings of *Rickettsia* in green lacewings, in the present study we sought to determine how frequently these insects carry rickettsial endosymbionts.

Lacewings belong to the Neuroptera, a small (~6000 species [16]) order of holometabolous insects, which also comprises, e.g., antlions (Myrmeleontidae) and owlflies (Ascalaphidae). This group is diverse in ecology and appearance, but is generally characterized by the predaceous lifestyle of larvae and adults of most species. Adults are characterized by two pairs of typically large, extensively veined wings [17]. Green lacewings (Chrysopidae) are probably the best known and studied family of this order, and are common across the globe, with the exception of Antarctica. Two aspects of green lacewing biology have especially attracted research interest in the past. One is the cryptic diversity and courtship songs of green lacewings. Several morphospecies of chrysopids comprise multiple reproductively isolated species, which often can be differentiated based on courtship song characteristics [18–20]. The second is the potential utility of green lacewings as biological control agents in crop environments [21]. Many species are efficient predators of aphids and other pests, and a few are mass-reared because of this quality [22–24].

We here report the first rickettsial symbiont screen of a representative sample of the European chrysopid fauna. We show that *Rickettsia* is a common symbiont of green lacewings, and that genetically diverse symbiont strains are associated with these insects. In light of the significant impacts of rickettsial and other endosymbionts on insects in general [25, 26], this study provides the first indication of a potentially important aspect of lacewing biology that has been neglected to date.

## Methods

### Animal collection and DNA extraction

The animals used in this study were collected with hand nets between 2009 and 2014 from a total of 38 locations in Germany (the majority of samples), Austria, France, Great Britain, Hungary, Portugal, and Mongolia. Sampling was focused on green lacewings (Chrysopidae), but also included one species each of brown lacewings (Hemerobiidae) and alderflies (Megaloptera, Sialidae). Where possible, for each species, we collected at least five individuals from multiple populations (i.e., locations). In total, we collected 103 individuals belonging to 18 species. A detailed list of sampled individuals can be found in Additional file 1: Table S1. All animals were either killed and stored in 80% EtOH, or first anaesthetized and killed with ethyl acetate, and later mounted and dried to facilitate determination. For *Rickettsia* screens, partial or complete abdomens were ground using sterile pestles, and digested using Proteinase K. DNA was then extracted using a chloroform/isoamyl alcohol protocol with subsequent ethanol precipitation, and stored in TE buffer at −20 °C.

### Screen for *Rickettsia* endosymbionts

DNA extracts were quality-checked via PCR by amplifying the mitochondrial cytochrome oxidase subunit I gene (*COI*), using the primers LCO1490 and HCO2198 [27]. PCR success was assessed visually by electrophoresis, and DNA extraction was repeated for samples in which PCR failed. All samples passing this check were then screened for *Rickettsia* using four diagnostic primer pairs, each specific for a single *Rickettsia* locus (*16S rRNA*, *atpA*, *coxA*, *gltA*, see [3] for primer sequences). All amplified *Rickettsia* PCR products were Sanger sequenced in both forward and reverse directions by an external service provider (GATC Biotech, Konstanz, Germany). Samples were considered as *Rickettsia* positive only when unambiguous, high quality *Rickettsia* sequences from any of the four loci were recovered. All sequences have been submitted to NCBI GenBank (see Additional file 2: Table S2 for accession numbers).

### Phylogenetic analyses

To facilitate phylogenetic classification of the *Rickettsia* strains associated with lacewings, one alignment was created for each of the four sequenced *Rickettsia* loci, and complemented with sequences from other *Rickettsia* strains previously characterized by Weinert et al. [3]. The sequences of each locus were aligned with MAFFT version 7.271 [28] using the ‘L-INS-i’ algorithm and concatenated to a supermatrix using FasConCat version 1.0 [29]. The final dataset consisted of 100 *Rickettsia* strains (32 from lacewings) and 3729 nucleotide positions in total. IQ-TREE version 1.5.3 [30] was then employed to 1) determine the best fitting nucleotide substitution models [31] and partitioning scheme using the ‘greedy’ algorithm [32], 2) perform a maximum likelihood tree search under this partitioning scheme. Clade credibility was assessed using 5000 ‘ultrafast’ bootstrap replicates [33]. Likewise, single gene trees were reconstructed for each of the four *Rickettsia* loci to identify potential recombination events. One such event was identified (Additional file 3: Figure S1), and the corresponding sample (‘*Chrysopa perla* CPer7’) removed from subsequent analyses. Finally, a host phylogeny based on mitochondrial COI was reconstructed. Sequences for samples ‘*Pseudomalla prasinus* DPan’ & ‘*Pseudomallada ventralis* 14278’ were generated using the COI primers mentioned above; all other sequences were taken from the NCBI nucleotide database. All trees were visualized using the online tool Evolview [34].

## Results

**Fig. 1:**
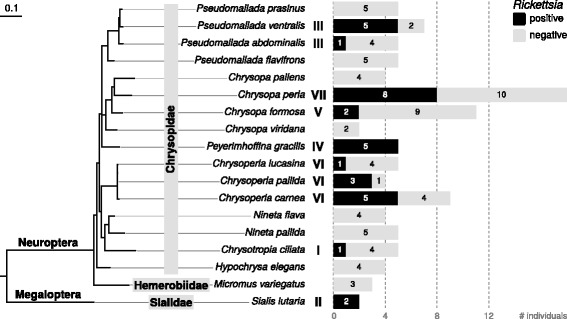
Result of *Rickettsia* screening in lacewings and allies. Maximum likelihood tree shows phylogenetic relationships of investigated species based on a 657 bp alignment of the mitochondrial cytochrome oxidase subunit 1 gene. For each of the species, a bar chart shows the number of sampled individuals, partitioned in *Rickettsia* positive individuals (*black part of bars*) and *Rickettsia* negative individuals (*grey part of bars*)

**Fig. 2:**
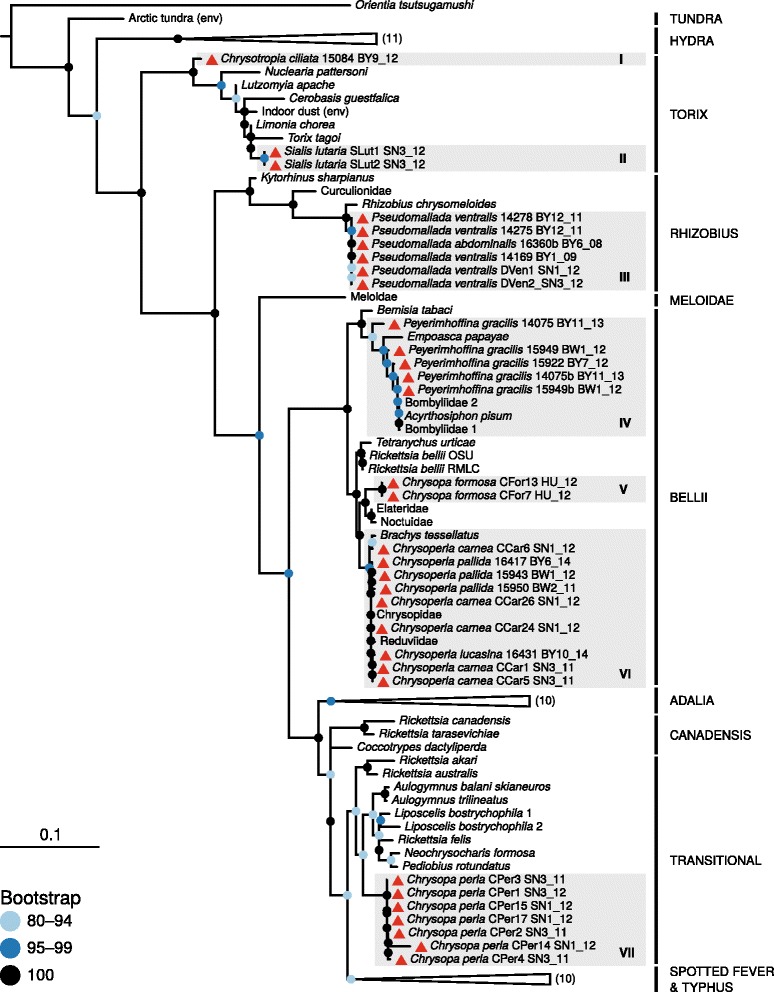
Maximum likelihood tree of *Rickettsia* strains based on four loci (3729 bp in total). Bootstrap support is color-coded as illustrate in the legend and based on 5000 replicates. *Rickettsia* group naming follows [3], as does naming of the strains. Briefly, most names correspond to *Rickettsia* hosts, and lowest available taxonomic rank is provided. Rickettsial species are named ‘*Rickettsia*‘ and environmental samples are labeled with ‘(env)’. For collapsed parts of the tree, the number of terminal nodes are parenthesized. Novel sequences are marked with red triangles and each is labeled with host species name, lab ID, and an abbreviation indicating sampling location and sampling date (separated with underscore). See Additional file 1: Table S1 for full information on all samples

In total, 103 individuals belonging to 18 species (Neuroptera: 16 Chrysopidae, 1 Hemerobiidae, Megaloptera :1 Sialidae) were screened, and *Rickettsia* was detected in 33 individuals from 10 species (Fig. 1). Within host species, *Rickettsia* prevalence ranged from 20 to 100% (Fig. 1), although these estimates stem mostly from small sample sizes (2–18). We performed a phylogenetic analysis of the detected strains together with previously characterized strains based on an incomplete matrix of four *Rickettsia* loci (*16S rRNA*, *atpA*, *coxA*, *gltA*), because not all loci were available for all previously characterized strains, and amplification failed for some of the loci from our strains as well (Additional file 2: Table S2). The tree recovered in our analysis is very similar to previous comprehensive analyses by Weinert et al. [3], and thus in agreement with their proposed classification scheme for *Rickettsia* species groups. The newly sequenced strains are found in four *Rickettsia* groups (‘Torix’, ‘Rhizobius’, ‘Bellii’, ‘Transitional’), and seven distinct lineages of rickettsial endosymbionts of lacewings can be differentiated (I–VII in Fig. 2).

All *Rickettsia* strains isolated from a single lacewing host species were very similar to each other based on the four sequenced loci, irrespective of when and where the individual was collected (Fig. 2). Mapping the presence of seven lineages onto the host phylogeny (Fig. 1) revealed that the presence of *Rickettsia* is not strongly associated with host phylogeny, i.e., it appears to be phylogenetically random. However, the presence of a particular *Rickettsia* lineage is not completely independent of host phylogeny. For example, the closely related *Chrysoperla carnea*, *C. lucasina* and *C. pallida* harbor nearly identical *Rickettsia* strains (Figs. 1 and 2).

## Discussion

The incidence of *Rickettsia* in lacewings reported here (32% of individuals, 44% of species) is considerably higher than that determined from other arthropod taxa via similar PCR screens [35]. It is, however, in line with a recent maximum likelihood estimation of overall *Rickettsia* incidence in terrestrial arthropods [1]. As other similar PCR based endosymbiont screens, our approach likely missed very low-titer *Rickettsia* infections and those that are found only in a small proportion of the tested species, and thus, the true *Rickettsia* incidence in lacewings may be even higher than that reported here. Disregarding the potentially undetected associations, *Rickettsia* can still be considered a common symbiont in green lacewings. This is true despite the seemingly small number of sampled species, which comprised around half of the German species of the small family Chrysopidae (16/29 species [36]).

Beyond their incidence, lacewings can also be considered as a hotspot for *Rickettsia* in terms of the genetic diversity of detected strains. With the exception of vectors for pathogenic *Rickettsia* (e.g., ticks, fleas) [37–39], no other arthropod host lineage shows a comparable diversity of *Rickettsia* strains. The strains found here belong to seven phylogenetically distinct lineages that are distributed widely across the known *Rickettsia* diversity (Fig. 2). This implies at least seven independent acquisitions of *Rickettsia* symbionts in lacewings. One potentially confounding effect for this estimate is homologous recombination. *Rickettsia* genomes typically harbor a recombination machinery, conjugation genes, and plasmids, all of which could contribute to genetic exchange between *Rickettsia* strains [40–43]. Within the dataset analyzed here, we found one clear case of recombination; based on *coxA* sequences, the *Chysopa perla* sample ‘CPer7’ clustered within the ‘Transitional’ group of *Rickettsia* strains (Additional file 3: Figure S1), as did all other strains from *Chrysopa perla* (Fig. 2). However, the 16S sequence of this strain was most similar to strains of the ‘Torix’ group (Additional file 3: Figure S1). We consequently excluded this sample from the supermatrix analysis. Of the seven distinct lineages of *Rickettsia* within lacewings, most are represented by multiple samples and/or loci, and recombination did thus likely not affect their phylogenetic placement. However, the sample *Chrysotropia ciliata* 15,084 was only represented by a single *16S* sequence in our dataset, and as the only member of *Rickettsia* lineage ‘I’ (Fig. 2), we cannot exclude the possibility that this lineage only appears to be distinct due to a recombination artifact.

Examples for other taxa frequently associated with *Rickettsia* include ladybirds [3, 14], spiders [44, 45], and water beetles of the genus *Deronectes* [46], as well as species in the dipteran families Dolichopodidae, Empidae, and Hyobotidae [47]. Strikingly, all of these taxa, and the chrysopids investigated here, are predaceous. Hence, the question arises whether our and previous reports of *Rickettsia* PCR positives represent true symbiotic associations, or rather contaminants from symbionts of prey taxa. However, multiple lines of evidence support the view that *Rickettsia* are vertically transmitted symbionts in lacewings: 1) The main prey of green lacewing larvae are aphids, and the low prevalence of *Rickettsia* in aphids [48] cannot account for the high incidence of this symbiont in lacewings. 2) *Rickettsia* strains from lacewings are genetically very diverse and host-specific (only a single *Rickettsia* strain per species, Fig. 2). It is very unlikely that individuals of a single species sampled at various locations and times acquired identical *Rickettsia* strains from their prey (or environment) by chance alone. A more parsimonious explanation is that the association of *Rickettsia* with lacewings is evolutionarily stable and pre-dates the geographic distribution of host populations sampled here. 3) We found that closely related lacewing species (e.g., *Chrysoperla* sp. & *Pseudomallada* sp.) harbor similar *Rickettsia* strains that are genetically distinct from other strains. Again, it is unlikely that such patterns arise by chance. Rather, we think it is best explained by the presence of a vertically transmitted *Rickettsia* symbiont in the last common ancestor of the recently diverged host species (Fig. 1).

*Rickettsia* is one of four symbionts reported from green lacewings to date. It has been recognized for nearly 50 years that some chrysopids are associated with symbiotic yeasts [49, 50]. Special morphological structures appear to have evolved in response to the presence of yeasts and are present only in lacewings that feed on pollen, nectar, and honeydew as adults, but not in species with predaceous adults [51]. It has consequently been suggested that symbiosis with yeasts is obligate for these lacewings, and that yeasts may provide essential nutrients for non-predaceous adult chrysopids [49]. However, evidence for nutritional supplementation is ambiguous [52] and the role of yeasts in lacewings remains unclear [53]. Furthermore, high densities of bacterial cells have been reported from microscopic investigations of the midgut of *Chrysoperla carnea*, potentially indicating a nutritional role for these bacteria [54]. However, it has also been suggested that midgut-associated bacteria in lacewings are transient, rather than symbiotic [55]. A “true” symbiont reported from lacewings is *Spiroplasma* (Mollicutes). Hayashi et al. detected this bacterium in a Japanese population of the chrysopid *Mallada desjardinsi* and further observed that *Spiroplasma* presence in mothers is strongly correlated with all-female broods [56]. *Spiroplasma* thus may act as a male-killer in this chrysopid, as has been reported previously from e.g., buprestid and ladybird beetles [5, 57]. The Hayashi group also detected *Rickettsia* in *Mallada desjardinsi*, but did not find evidence for sex-ratio distortion in this green lacewing species [56].

These examples illustrate how symbionts can profoundly impact lacewing biology. While past research has focused on the role of yeasts, the incidence and diversity of the bacterial symbiont *Rickettsia* as reported here warrant investigations of its effects in lacewings. This is not only interesting from evolutionary and ecological perspectives, but also important for the application of green lacewings in biological pest control. Larval chrysopids are efficient predators of aphids, psyllids, whiteflies, and other crop pests, and a number of species are currently used as biological control agents [58]. Many traits that are typically encoded by symbionts could potentially impact the effectiveness of chrysopids in that respect: nutritional supplementation, reproductive manipulations or protection from viruses and parasitoids. Understanding the interactions of *Rickettsia* and other symbionts with lacewings would therefore facilitate a targeted manipulation of the biological properties of lacewings, and thus improve their value in biological control.

## Conclusion

*Rickettsia* are common symbionts of green lacewings (Chrysopidae). While the importance of these symbiotic associations remain to be determined, the large genetic diversity of *Rickettsia* in lacewings, species-specific distributions, and previous reports on symbiont-mediated sex ratio distortion suggest they are relevant to lacewing biology. Several green lacewing species are being reared efficiently in large numbers for the purpose of biological pest control. These species are clear candidates for use in investigating the characteristics of rickettsial symbiosis in lacewings.

## Additional files

Additional file 1: Table S1. Sampling information for all individuals investigated in this study. (ODS 26 kb)

Additional file 2: Table S2. NCBI accession numbers for all sequences created in this study. Missing accession numbers indicate that PCR was not successful for corresponding fragment. (ODS 24 kb)

Additional file 3: Figure S1. Recombination in Rickettsia from lacewings. Maximum likelihood trees for both CoxA and 16S rRNA are shown. Presumed recombinant Rickettsia strain is highlighted with an arrow in both trees. (PDF 62 kb)

## References

[1] Weinert LA, Araujo-Jnr EV, Ahmed MZ, Welch JJ (2015). The incidence of bacterial endosymbionts in terrestrial arthropods. Proc R Soc B.

[2] Parola P, Paddock CD, Raoult D (2005). Tick-borne rickettsioses around the world: emerging diseases challenging old concepts. Clin Microbiol Rev.

[3] Weinert LA, Werren JH, Aebi A, Stone GN, Jiggins FM (2009). Evolution and diversity of *Rickettsia* bacteria. BMC Biol.

[4] Perlman SJ, Hunter MS, Zchori-Fein E (2006). The emerging diversity of *Rickettsia*. Proc R Soc B.

[5] Lawson ET, Mousseau TA, Klaper R, Hunter MD, Werren JH (2001). *Rickettsia* associated with male-killing in a buprestid beetle. Heredity.

[6] Hurst GD, Graf von der Schulenburg JH, Majerus TM, Bertrand D, Zakharov IA, Baungaard J (1999). Invasion of one insect species, *Adalia bipunctata*, by two different male-killing bacteria. Insect Mol Biol.

[7] Giorgini M, Bernardo U, Monti MM, Nappo AG, Gebiola M (2010). *Rickettsia* symbionts cause parthenogenetic reproduction in the parasitoid wasp *Pnigalio soemius* (hymenoptera: Eulophidae). Appl Environ Microbiol.

[8] Hagimori T, Abe Y, Date S, Miura K (2006). The first finding of a *Rickettsia* bacterium associated with parthenogenesis induction among insects. Curr Microbiol.

[9] Himler AG, Adachi-Hagimori T, Bergen JE, Kozuch A, Kelly SE, Tabashnik BE (2011). Rapid spread of a bacterial symbiont in an invasive whitefly is driven by fitness benefits and female bias. Science.

[10] Cass BN, Himler AG, Bondy EC, Bergen JE, Fung SK, Kelly SE (2015). Conditional fitness benefits of the *Rickettsia* bacterial symbiont in an insect pest. Oecologia.

[11] Lukasik P, Guo H, van Asch M, Ferrari J, Godfray HCJ. Protection against a fungal pathogen conferred by the aphid facultative endosymbionts *Rickettsia* and *Spiroplasma* is expressed in multiple host genotypes and species and is not influenced by co-infection with another symbiont. J Evol Biol. 2013;26:2654–61. 10.1111/jeb.12260 24118386

[12] Hendry TA, Hunter MS, Baltrus DA (2014). The facultative symbiont *Rickettsia* protects an invasive whitefly against entomopathogenic *Pseudomonas syringae* strains. Appl Environ Microbiol.

[13] Cowdry EV (1923). The distribution of *Rickettsia* in the tissues of insects and arachnids. J Exp Med.

[14] Weinert LA, Tinsley MC, Temperley M, Jiggins FM (2007). Are we underestimating the diversity and incidence of insect bacterial symbionts? A case study in ladybird beetles. Biol Lett.

[15] Gerth M, Saeed A, White JA, Bleidorn C (2015). Extensive screen for bacterial endosymbionts reveals taxon-specific distribution patterns among bees (hymenoptera, Anthophila). FEMS Microbiol Ecol.

[16] Grimaldi D, Engel MS. Evolution of the insects. Cambridge: Cambridge University Press; 2005.

[17] New TR. Handbuch der Zoologie, Band 4: Arthropoda, 2 Hälfte: Insecta, Teilband 30, Planipennia. Berlin: DeGruyter; 1989.

[18] Henry CS, Wells MM, Pupedis RJ (1993). Hidden taxonomic diversity within *Chrysoperla plorabunda* (Neuroptera: Chrysopidae): two new species based on courtship songs. Ann Entomol Soc Am.

[19] Henry CS (1985). Sibling species, call differences, and speciation in green lacewings (Neuroptera: Chrysopidae: *Chrysoperla*). Evolution.

[20] Henry CS (1983). Acoustic recognition of sibling species within the holarctic lacewing *Chrysoperla carnea* (Neuroptera: Chrysopidae). Syst Entomol.

[21] McEwen PK, New TR, Whittington AE. Lacewings in the crop environment. Cambridge: Cambridge University Press; 2007.

[22] Ridgway RL, Morrison RK, Badgley M (1970). Mass rearing a green lacewing. J Econ Entomol.

[23] Tauber MJ, Tauber CA, Daane KM, Hagen KS (2000). Commercialization of predators: recent lessons from green lacewings (Neuroptera: Chrysopidae: Chrysoperla). Am Entomol.

[24] Albuquerque GS, Tauber CA, Tauber MJ (1994). *Chrysoperla externa* (Neuroptera: Chrysopidae): life history and potential for biological control in central and South America. Biol Control.

[25] Douglas AE. Multiorganismal insects: diversity and function of resident microorganisms. Annu Rev Entomol. 2014;60:17–34. 10.1146/annurev-ento-010814-020822 PMC4465791 25341109

[26] Wernegreen JJ (2012). Endosymbiosis. Curr Biol.

[27] Folmer O, Black M, Hoeh W, Lutz R, Vrijenhoek R (1994). DNA primers for amplification of mitochondrial cytochrome c oxidase subunit I from diverse metazoan invertebrates. Mol Mar Biol Biotechnol.

[28] Katoh K, Standley DM (2013). MAFFT multiple sequence alignment software version 7: improvements in performance and usability. Mol Biol Evol.

[29] Kück P, Meusemann K (2010). FASconCAT: convenient handling of data matrices. Mol Phylogenet Evol.

[30] Nguyen L-T, Schmidt HA, von Haeseler A, Minh BQ (2015). IQ-TREE: a fast and effective stochastic algorithm for estimating maximum-likelihood phylogenies. Mol Biol Evol.

[31] Kalyaanamoorthy S, Minh BQ, Wong TKF, von Haeseler A, Jermiin LS. ModelFinder: fast model selection for accurate phylogenetic estimates. Nat Methods. 2017;14:587–89. 10.1038/nmeth.4285 PMC5453245 28481363

[32] Lanfear R, Calcott B, Kainer D, Mayer C, Stamatakis A (2014). Selecting optimal partitioning schemes for phylogenomic datasets. BMC Evol Biol.

[33] Minh BQ, Nguyen MAT, von Haeseler A (2013). Ultrafast approximation for phylogenetic bootstrap. Mol Biol Evol.

[34] He Z, Zhang H, Gao S, Lercher MJ, Chen W-H, Hu S (2016). Evolview v2: an online visualization and management tool for customized and annotated phylogenetic trees. Nucleic Acids Res.

[35] Duron O, Bouchon D, Boutin S, Bellamy L, Zhou L, Engelstädter J (2008). The diversity of reproductive parasites among arthropods: *Wolbachia* do not walk alone. BMC Biol.

[36] Saure C, Klausnitzer B (2003). Verzeichnis der Netzflügler (Neuroptera) Deutschlands. Entomofauna Germanica 6.

[37] Parola P, Davoust B, Raoult D (2005). Tick- and flea-borne rickettsial emerging zoonoses. Vet Res.

[38] Azad A (1998). Rickettsial pathogens and their arthropod vectors. Emerg Infect Dis.

[39] Aspöck H (2008). Durch Arthropoden übertragene Erreger von Infektionen des Menschen in Mitteleuropa--ein Update. Mitt Dtsch Ges Allg Angew Entomol.

[40] Gillespie JJ, Joardar V, Williams KP, Driscoll T, Hostetler JB, Nordberg E (2011). A *Rickettsia* genome overrun by mobile genetic elements provides insight into the acquisition of genes characteristic of an obligate intracellular lifestyle. J Bacteriol.

[41] Andersson SG, Zomorodipour A, Andersson JO, Sicheritz-Pontén T, Alsmark UC, Podowski RM (1998). The genome sequence of *Rickettsia prowazekii* and the origin of mitochondria. Nature.

[42] Ogata H, Renesto P, Audic S, Robert C, Blanc G, Fournier P-E (2005). The genome sequence of *Rickettsia felis* identifies the first putative conjugative plasmid in an obligate intracellular parasite. PLoS Biol.

[43] Weinert LA, Welch JJ, Jiggins FM (2009). Conjugation genes are common throughout the genus *Rickettsia* and are transmitted horizontally. Proc R Soc B.

[44] Ceccarelli FS, Haddad CR, Ramírez MJ (2016). Endosymbiotic Rickettsiales (Alphaproteobacteria) from the spider genus *Amaurobioides* (Araneae: Anyphaenidae). J Arachnol.

[45] Goodacre SL, Martin OY, Thomas CFG, Hewitt GM (2006). *Wolbachia* and other endosymbiont infections in spiders. Mol Ecol.

[46] Küchler SM, Kehl S, Dettner K (2009). Characterization and localization of *Rickettsia* sp. in water beetles of genus *Deronectes* (Coleoptera: Dytiscidae). FEMS Microbiol Ecol.

[47] Martin OY, Puniamoorthy N, Gubler A, Wimmer C, Bernasconi MV (2013). Infections with *Wolbachia*, *Spiroplasma*, and *Rickettsia* in the Dolichopodidae and other Empidoidea. Infect Genet Evol.

[48] Oliver KM, Degnan PH, Burke GR, Moran NA (2010). Facultative symbionts in aphids and the horizontal transfer of ecologically important traits. Annu Rev Entomol.

[49] Hagen KS, Tassan RL. Exploring nutritional roles of extracellular symbiotes on the reproduction of honeydew feeding adult chrysopids and tephritids. In: Rodriguez JG, editor. Insect and mite nutrition. Amsterdam: North-Holland Pub. Co; 1972. p. 323–351.

[50] Hagen KS, Tassan RL, Sawall EF (1970). Some ecophysiological relationships between certain *Chrysopa*, honeydews and yeasts. Bollettino del Laboratorio di Entomologia Agraria Filippo Silvestri.

[51] Woolfolk SW, Cohen AC, Inglis GD (2004). Morphology of the alimentary canal of *Chrysoperla rufilabris* (Neuroptera: Chrysopidae) adults in relation to microbial symbionts. Ann Entomol Soc Am.

[52] Gibson CM, Hunter MS (2005). Reconsideration of the role of yeasts associated with *Chrysoperla* green lacewings. Biol Control.

[53] Albuquerque G, Tauber C, Tauber M. Green lacewings (Neuroptera: Chrysopidae). In: Antônio Ricardo Panizzi JRPP, editor. Insect bioecology and nutrition for integrated pest management. Boca Raton: CRS Press; 2012. p. 593–631.

[54] Chen T-Y, Chu C-C, Hu C, Mu J-Y, Henneberry TJ (2006). Observations on midgut structure and content of *Chrysoperla carnea* (Neuroptera: Chrysopidae). Ann Entomol Soc Am.

[55] Woolfolk SW, Douglas IG (2004). Microorganisms associated with field-collected *Chrysoperla rufilabris* (Neuroptera: Chrysopidae) adults with emphasis on yeast symbionts. Biol Control.

[56] Hayashi M, Watanabe M, Yukuhiro F, Nomura M, Kageyama D (2016). A nightmare for males? A maternally transmitted male-killing bacterium and strong female bias in a green lacewing population. PLoS One.

[57] Hurst GDD, Majerus MEN, Walker LE (1992). Cytoplasmic male killing elements in *Adalia bipunctata* (Linnaeus) (Coleoptera: Coccinellidae). Heredity.

[58] Senior LJ, McEwen PK. The use of lacewings in biological control. In: P. K. McEwen and T. R. New and A. E. Whittington, editor. Lacewings in the Crop Environment. Cambridge: Cambridge University Press; 2001. p. 296–302.

