# Supplementary material for: Green lacewings (Neuroptera: Chrysopidae) are commonly associated with a diversity of rickettsial endosymbionts
Source: Zoological Lett. 2017 Aug 14;3:12. doi: 10.1186/s40851-017-0072-9 (PMC5557424; doi:10.1186/s40851-017-0072-9)
Supplement: Supplementary file 3 — Recombination in Rickettsia from lacewings. Maximum likelihood trees for both CoxA and 16S rRNA are shown. Presumed recombinant Rickettsia strain is highlighted with an arrow in both trees. (PDF 62 kb) [file 40851_2017_72_MOESM3_ESM.pdf]

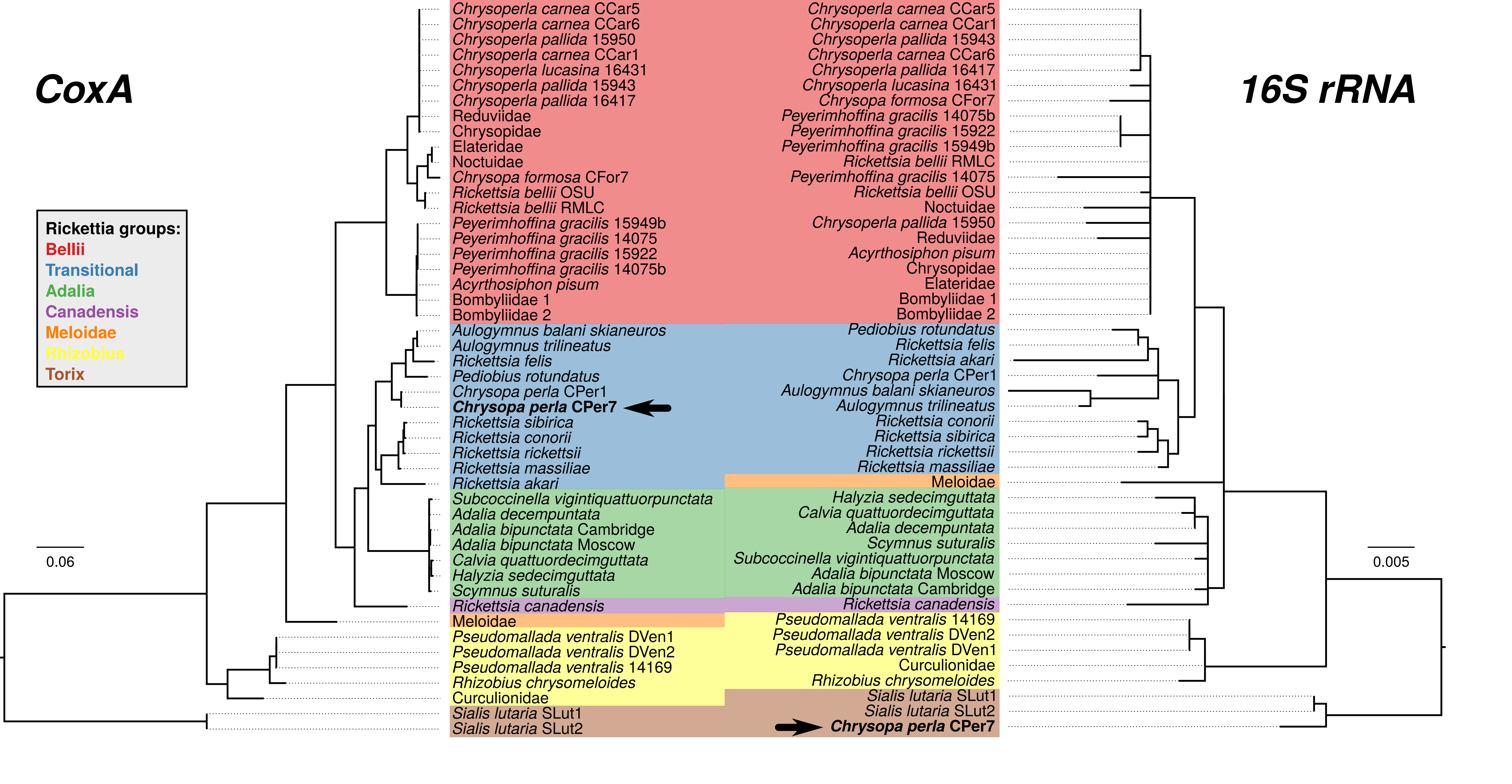

CoxA
16S rRNA
Rickettsia groups:
Bellii
Transitional
Adalia
Canadensis
Meloidae
Rhizobius
Torix
0.06
0.005
Chrysoperla carnea CCar5
Chrysoperla carnea CCar6
Chrysoperla pallida 15950
Chrysoperla carnea CCar1
Chrysoperla lucasina 16431
Chrysoperla pallida 15943
Chrysoperla pallida 16417
Reduviidae
Chrysopidae
Elateridae
Noctuidae
Chrysopa formosa CFor7
Rickettsia bellii OSU
Rickettsia bellii RMLC
Peyerimhoffina gracilis 15949b
Peyerimhoffina gracilis 14075
Peyerimhoffina gracilis 15922
Peyerimhoffina gracilis 14075b
Acyrthosiphon pisum
Bombyliidae 1
Bombyliidae 2
Aulogymnus balani skianeuros
Aulogymnus trilineatus
Rickettsia felis
Pediobius rotundatus
Chrysopa perla CPer1
Chrysopa perla CPer7
Rickettsia sibirica
Rickettsia conorii
Rickettsia rickettsii
Rickettsia massiliae
Rickettsia akari
Subcoccinella vigintiquattuorpunctata
Adalia decempuntata
Adalia bipunctata Cambridge
Adalia bipunctata Moscow
Calvia quattuordecimguttata
Halyzia sedecimguttata
Scymnus suturalis
Rickettsia canadensis
Meloidae
Pseudomallada ventralis DVen1
Pseudomallada ventralis DVen2
Pseudomallada ventralis 14169
Rhizobius chrysomeloides
Curculionidae
Sialis lutaria SLut1
Sialis lutaria SLut2
Chrysoperla carnea CCar5
Chrysoperla carnea CCar1
Chrysoperla pallida 15943
Chrysoperla carnea CCar6
Chrysoperla pallida 16417
Chrysoperla lucasina 16431
Chrysopa formosa CFor7
Peyerimhoffina gracilis 14075b
Peyerimhoffina gracilis 15922
Peyerimhoffina gracilis 15949b
Rickettsia bellii RMLC
Peyerimhoffina gracilis 14075
Rickettsia bellii OSU
Noctuidae
Chrysoperla pallida 15950
Reduviidae
Acyrthosiphon pisum
Chrysopidae
Elateridae
Bombyliidae 1
Bombyliidae 2
Pediobius rotundatus
Rickettsia felis
Rickettsia akari
Chrysopa perla CPer1
Aulogymnus balani skianeuros
Aulogymnus trilineatus
Rickettsia conorii
Rickettsia sibirica
Rickettsia rickettsii
Rickettsia massiliae
Meloidae
Halyzia sedecimguttata
Calvia quattuordecimguttata
Adalia decempuntata
Scymnus suturalis
Subcoccinella vigintiquattuorpunctata
Adalia bipunctata Moscow
Adalia bipunctata Cambridge
Rickettsia canadensis
Pseudomallada ventralis 14169
Pseudomallada ventralis DVen2
Pseudomallada ventralis DVen1
Curculionidae
Rhizobius chrysomeloides
Sialis lutaria SLut1
Sialis lutaria SLut2
Chrysopa perla CPer7
